# Gamification in mHealth Apps for Rehabilitation: Protocol for a Scoping Review

**DOI:** 10.2196/63600

**Published:** 2025-04-28

**Authors:** Jacqueline Dawson, Randall Nee, Christian Ramirez, Sharlene Reyes, David Sanchez, Tulsi Sukhadia, Andrew Bartlett

**Affiliations:** 1 Department of Physical Therapy California State University, Long Beach Long Beach, CA United States; 2 Department of Physical Therapy California State University, Northridge Northridge, CA United States

**Keywords:** telerehabilitation, remote physical therapy, home exercise program, digital health, mHealth, mobile health, rehabilitation, scoping review, gamification, mobile health app

## Abstract

**Background:**

The use of gamification in physical therapy mobile health interventions has increased rapidly in recent years, particularly with self-management of exercise in a home environment. Prior research has focused broadly on digital exergames, such as virtual reality or video games, or specifically on single concerns, such as stroke or musculoskeletal rehabilitation.

**Objective:**

This scoping review aims to identify studies that have implemented gamification in rehabilitative interventions through mobile apps to treat all conditions addressed by physical therapists. Characteristics related to the rehabilitative process, gamification elements, and patient-related outcomes will be examined.

**Methods:**

A literature search will be conducted on the following databases: MEDLINE (Ovid), Embase, CINAHL, PeDRO, Scopus, and Web of Science. Study inclusion criteria will be based on the PICO (Population, Intervention, Comparison, Outcome) framework, with publications describing the use of a gamified mobile app in a movement-based intervention in any area of physical therapy included. The reporting of results will adhere to PRISMA-ScR (Preferred Reporting Items for Systematic Reviews and Meta-Analyses Extension for Scoping Reviews). A narrative synthesis of included publications will be performed.

**Results:**

Database searches were completed in May 2024 and yielded 2148 publications, with an additional 49 records identified through manual searching of references. Title and abstract screening, full-text screening, and data extraction are expected to be completed by April 2025. The review is expected to be completed by September 2025.

**Conclusions:**

Findings from this scoping review will provide evidence on gamified mobile apps to assist physical rehabilitation professionals with decision-making on remote interventions. Understanding game elements used in rehabilitative mobile apps may enhance patient engagement and adherence, which may ultimately improve patient-related outcomes.

**Trial Registration:**

Open Science Framework fz9nq; https://osf.io/fz9nq

## Introduction

Physical rehabilitation interventions following injury or impairment can improve the quality of life through movement-based care [1]. Physical therapists are trained to deliver interventions that treat a multitude of health conditions, including musculoskeletal, neurological, cardiopulmonary, cancer, metabolic, and pain issues. The impact of physical therapy can extend beyond the individual to the societal level, especially when the rehabilitation is comprehensive [2]. Thus, physical therapy is not only long-lasting and life-changing but also economically beneficial [3].

Physical rehabilitation is a long-term process that can begin in an inpatient setting and require subsequent services in the outpatient environment. Yet, for therapy to be effective, ongoing exercise in the home environment is often needed [4]. Home exercise programs (HEPs) are forms of supplemental treatment given to individuals to promote progression toward goals and maintain improvements outside of supervised therapy. However, patient adherence, or the extent to which an individual’s behavior corresponds to provider recommendations, has been identified as the most significant, yet most unpredictable variable factor in HEP effectiveness [5].

Patient adherence to an HEP is influenced by many factors, from the individuals’ beliefs and attitudes toward the treatment to self-efficacy and motivation in performing the activity [5]. Adherence to HEPs may be high in the early stages of treatment but tends to diminish over time, with typical HEP adherence rates estimated to be as low as 30% [4,6]. Given that adherence to HEPs is directly associated with improvement in clinical outcomes [6], strategies to increase adherence are needed to bridge the gap between performance in clinical and home settings. Technological advancements such as mobile health (mHealth) have been proposed to support self-management in home-based rehabilitative programs through low-cost scalable solutions [7]. mHealth is defined as any mobile device, such as smartphones or tablets, or app used to implement medical and public health practice with patients in a home setting through two-way communication [8]. Of the various forms of mHealth, mobile apps targeting health and wellness have seen substantial growth. To date, there are over 350,000 health-related apps available through various app stores worldwide, with nearly 14 million app downloads recorded in 2024 alone [9,10]. While these apps vary widely in terms of functionality, most health-related apps incorporate features such as remote monitoring, exercise instruction, activity tracking, and goal setting [11].

One mobile app characteristic that is important to consider in the context of rehabilitation adherence is gamification, or the use of game design elements to increase patient motivation and engagement [12]. In therapy, gamification has been explored primarily in pediatric neurorehabilitation to focus attention and facilitate repetition through hands-on immersive gameplay [13]. With mobile apps specifically, there has been rapid growth in the use of gamification to track and promote physical activity, such as with the popular augmented reality game, Pokémon GO, which increased daily step counts by 25% in over 32,000 users without directly promoting health outcomes [14]. However, the use of gamelike elements in rehabilitative mHealth apps is still emerging, and evidence supporting its therapeutic benefit is unclear.

Several reviews have examined the use of gamification in rehabilitation [15-20]. However, no review has focused solely on gamification for rehabilitative interventions implemented through mobile apps across all conditions treated by physical therapists. Adlakha et al [15], Alfieri et al [16], and Berton et al [17] considered gamification in rehabilitation without a focus on mobile apps [15-17], while Nussbaum et al [18] examined mobile apps in rehabilitation without a focus on gamification. Other reviews have focused on gamified mobile apps for specific conditions, such as stroke [19] and shoulder [20] rehabilitation, while Ryan et al [11] performed a content analysis of rehabilitation mHealth apps for all conditions and reported the prevalence of features and outcomes.

Given the growing popularity of gamified mobile apps in rehabilitative HEPs and the limited research examining their evidence, a review of gamified mHealth apps across all health conditions treated by physical therapists is warranted. The objective of this scoping review is to summarize the available information from clinical studies that have used game design elements in mobile apps for the exercise-based rehabilitation of patients in physical therapy. We will map and evaluate evidence according to 3 categories [20]: (1) rehabilitative process, (2) gamification elements, and (3) patient-related outcomes.

## Methods

### Study Design

The scoping review will be conducted using the framework by Arskey and O’Malley [21], which includes the following five stages: (1) study aim identification, (2) relevant study identification, (3) study selection, (4) data extraction, and (5) synthesis and reporting of research results. An optional sixth stage, stakeholder consultation, will be considered. The study inclusion criteria will be based on the PICO (Population, Intervention, Comparison, Outcome) framework [22]. However, the comparison will not be considered in this review. For the reporting of results, we will adhere to PRISMA-ScR (Preferred Reporting Items for Systematic Reviews and Meta-Analyses Extension for Scoping Reviews) [23].

### Stage 1: Study Aim Identification

This scoping review aims to synthesize current research on gamified mHealth apps used in physical therapy rehabilitation. Specifically, this review attempts to answer the following questions:

- Rehabilitative process
  What health conditions treated by physical therapy have used gamified mobile apps?
  In what phase of the rehabilitation process is the gamified mobile app used?
- Gamification elements
  How is gamification implemented in the rehabilitative intervention?
  What game design elements are used?
- Outcomes
  Which patient-related health, adherence, and safety outcomes are evaluated?
  Which user experience–related outcomes were evaluated?

### Stage 2: Identification of Relevant Studies

#### Overview

Researchers used scoping searches to identify keywords and synonyms to include in the search strategy (Table 1). A draft search strategy was developed for MEDLINE (Ovid) with the assistance of a research librarian, who helped translate keywords into controlled subject headings (Multimedia Appendix 1).

**Table 1:** Keyword and synonym search strategy.

Keyword	Synonyms
Rehabilitation	Telerehabilitation, telehealth, physical therapy, physiotherapy, preoperative care, postoperative care, patient education, disease management
Exercise therapy	Physical therapy modalities, therapeutic exercise, physical training, physical exercise, resistance training, exercise therapy, kinesiology, kinesiotherapy, aerobic exercise
Video games	Exergame, serious game, virtual reality, augmented reality, mobile health, mHealth, eHealth, remote, wearable, web, digital

Researchers adapted the search strategies for the following electronic databases MEDLINE (Ovid), Embase, CINAHL, PeDRO, Scopus, and Web of Science. Searches were conducted from inception to May 2024 and resulted in 2148 studies. For studies that met the inclusion criteria, references were also evaluated for potential inclusion, identifying an additional 49 studies. Only studies written in English with full-text articles were considered. No gray literature was designated for searching.

#### Inclusion Criteria

Regarding *population*, publications centered on patients in rehabilitative clinical studies with any condition and at any stage of care will be included. Regarding *intervention*, publications will be included that describe the use of a mobile app with gamification elements used in a movement-based intervention in any area of physical therapy. Physical therapy interventions that fit these criteria include therapeutic exercises, functional training, health and wellness promotion, respiratory exercises, motor skill training, or exercise education [1]. Regarding *outcomes*, the following patient-related outcomes will be included: measures of clinical effectiveness (eg, pain intensity, motor function), functional outcomes that enable independence (eg, gait speed, balance), physical or exercise capacity (eg, muscle strength, aerobic fitness), adherence, and adverse events. User experience–related outcomes will also be recorded, including usability (eg, error rate, ease of use) and acceptability (eg, motivation, enjoyment).

#### Exclusion Criteria

Articles will be excluded if the intervention is not rehabilitation-related or if the intervention is not provided by a physical therapist or under remote supervision by a physical therapist (eg, self-directed physical activity intervention for fitness outcomes). Further, articles will be excluded if movement- or exercise-based therapies are not used, such as manual therapy, respiratory techniques, electrotherapy, or physical agents. Studies will also be excluded if they do not use gamification and a mobile app to deliver the intervention. For example, interventions that solely use other forms of technology such as texting, videoconferencing, computer programs, video game consoles, robotics, exoskeletons, orthoses, or sensors will be cause for exclusion. Lastly, an article will be excluded if it is a review (eg, systematic or nonsystematic review), meta-analysis, case study or series, abstract, thesis, dissertation, commentary, opinion paper, or protocol paper.

### Stage 3: Study Selection

Records retrieved from the search will be managed through CADIMA (Julius Kuhn-Institut), where any duplicate records will be removed before screening. Two rounds of screening will be performed: (1) title and abstract screening and (2) full-text review. Reviewers will undergo training and practice screening 5% of the search results before the screening of all search results. Title and abstract screening will be performed by 2 reviewers who will independently conduct screening on the same articles using the inclusion and exclusion criteria. Any discrepancies will be resolved by a third reviewer (JD). Articles included in the title/abstract screening round will advance to a full-text review. Two reviewers will conduct a full-text review independently of each other on the same articles. Any discrepancies in article inclusion will be discussed and, if necessary, resolved by a third reviewer (JD). For an article to be excluded, all reviewers must agree. Reasons for full-text exclusion will be noted and reported in the scoping review.

### Stage 4: Data Extraction

Data extraction will be performed in two stages. In the first stage, primary data will be extracted using a standardized form by two researchers working independently and in parallel. Extraction fields will be developed from the preliminary search and designed to address the research questions. The data extraction form (Multimedia Appendix 2) collects bibliographical information, including authors, date of publication, and country of publication; participant characteristics, including condition addressed, intervention setting, and phase of rehabilitation; study design, including study type and blinding; exercise intervention characteristics, including level of supervision, frequency, intensity, duration, and type of exercise; technology characteristics, including type of delivery, intended audience, field of application, and game components; and outcomes reported, including outcome types, measures, and results. In the second stage, the methodological quality of the trials will be assessed using the 11-item PEDro scale [24]. For consistency and error reduction, one reviewer (JD) will oversee the data extraction process. For eligible studies that require clarification of methods or have missing data, the corresponding author will be contacted.

### Stage 5: Collating, Summarizing, and Reporting the Results

The fifth stage will involve data analysis and synthesis of the results in relation to the research question. The research questions will be used to organize the analysis of conditions, interventions, and outcomes from data collected on the extraction form. For conditions, studies will be classified into the following categories as done in a previous review [18]: cancer, cardiac, general rehabilitation, musculoskeletal, other neurologic diseases, pulmonary diseases, spinal cord injury, stroke, and traumatic brain injury. For interventions, a content analysis will be performed to identify themes within the interventions for each condition. In addition, frequency counts will be used to analyze the characteristics of interventions and outcome measures for each condition.

Results will be summarized qualitatively in narrative form, with the use of tables and diagrams where appropriate. Findings will be reported to answer each of the research questions and will therefore describe health conditions and rehabilitation phases addressed with gamified mobile apps; study design, intervention characteristics, and game elements used for each condition; and patient-reported outcomes, adherence, safety, and usability measures.

The discussion of findings will be structured based on identified themes and whether the research questions were answered. In addition, gaps in knowledge and implications for future research will be discussed.

### Stage 6: Stakeholder Consultation

The last stage is an optional step intended to inform key stakeholders and validate study findings [21]. We will aim to engage relevant stakeholders, including physical therapists, mobile app developers, and rehabilitative researchers. Consultations will be held to inform data interpretation and results dissemination.

## Results

**Figure 1:**
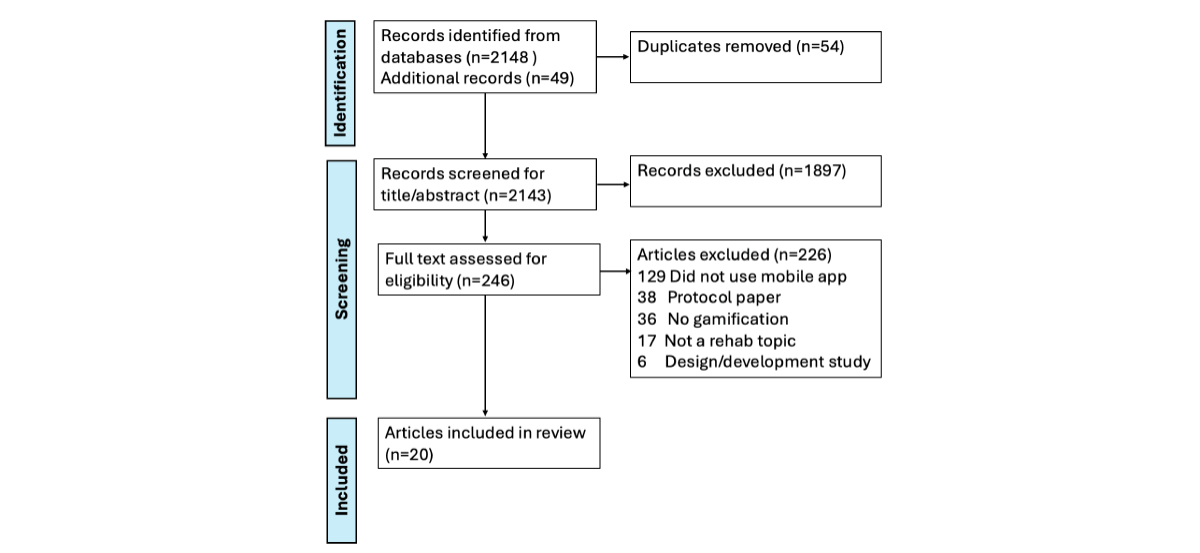
PRISMA (Preferred Reporting Items for Systematic Reviews and Meta-Analyses) flow diagram for selection of studies used in the analysis.

This scoping review was registered with the Open Science Framework on June 4, 2024. Electronic database searches were completed in May 2024 before abstract and title screening, resulting in 2148 studies identified from inception to 2024 (Figure 1). An additional 49 studies were identified from manual searching of references. Of these identified studies, 54 duplicates were removed, 1897 were excluded during title/abstract screening, and 226 were excluded during full-text screening. A total of 20 articles were therefore selected for full-text analysis. Data extraction and analysis are expected to be completed by June 2025. Results will be presented in narrative, graphical, and tabular representations to answer each of the research questions. Findings will therefore be structured to emphasize the use of gamified mobile apps by health condition, rehabilitation phase, intervention, and patient-reported outcomes. Additionally, adherence, safety, and usability measures will be presented.

## Discussion

This scoping review will map studies related to conditions treated by physical therapists in relation to the use of gamification and intervention delivery through mobile apps. The ubiquity of mobile devices and the capability of current technology make mobile apps an ideal tool for providing scalable low-cost HEPs to bridge care from the clinic to the home environment. Gamified mobile apps can be an attractive option for patients to maintain their rehabilitation routine as they provide high motivation and adherence, which in turn, can influence functional outcomes. However, the sheer number of available apps makes selection challenging for clinicians and patients alike. Although Ryan et al [11] reported on the prevalence of rehabilitative mHealth apps in app stores and their features, and several reviews have examined the use of gamification in rehabilitation in specific conditions [15-20], no review has focused solely on gamification for rehabilitative interventions implemented through mobile apps across all conditions treated by physical therapists. Thus, aggregation of the available evidence is needed to identify knowledge gaps and synthesize the usefulness of gamified mobile apps in the treatment and self-management of patients receiving physical rehabilitation. Findings from this scoping review will help inform the design of future studies to improve unmet medical needs and ultimately optimize patient outcomes.

This review has several strengths. It is the first review of gamified mobile apps that is inclusive of all conditions treated by physical therapists and may therefore provide a new perspective. Prior reviews on physical rehabilitation have captured narrower topics, such as focusing on one condition, gamification only, or mobile apps only. Expanding the review across all conditions can give insight into areas that are most frequently addressed, areas that are emerging, and areas of digital practice that are understudied. Another strength of this review is its focus on implications for clinical practice. Prior reviews surrounding mobile apps have described technology features and means by which mHealth is implemented in physical therapy interventions. However, none have focused on patient-reported outcomes and how this may translate to clinical practice. Finally, we will explore the methodological quality of the studies using the PEDRo scale, which was developed for physical therapy interventions. Although interpretation of the PEDRo scale is not to be taken as a measure of validity, it may provide insight into the clinical usefulness of this review and highlight areas where future research is needed.

### Limitations

One limitation of this scoping review would be the inability to access the full text of all included studies. To counter this limitation, we will make the best effort to contact authors and report the outcomes of the communication (eg, how many authors respond, send data). A second limitation is the exclusion of gray literature, as unpublished theses, dissertations, conference proceedings, abstracts only, opinion papers, and commentaries will not be considered. Reviewers and extractors will also not be blinded to the journal names or authors during screening or from the research questions during data extraction, both of which would minimize the risk of bias. This decision was made for the pragmatic consideration of completing the screening and extraction of studies promptly. Lastly, we will not conduct a formal evaluation of the risk of bias through a tool such as Cochrane’s Risk of Bias. This is in line with the goal of a scoping review to provide an overview of the existing publications rather than critically appraise each source of evidence.

### Conclusions

With the use of digital health and telerehabilitation on the rise, the topic of mHealth apps for physical therapy interventions is an emerging and important topic for clinicians. This scoping review seeks to map existing literature on the use of gamified mobile apps in physical rehabilitation in a home environment. Results will summarize research on gamified mobile apps to assist physical rehabilitation professionals with decision-making on remote interventions to enhance patient engagement and adherence. This will help identify knowledge gaps and advance rehabilitative mHealth research, which may ultimately improve patient-related outcomes.

## Appendix

Multimedia Appendix 1 Search strategy.

Multimedia Appendix 2 Data collection form.

Multimedia Appendix 3 PRISMA-P checklist.

## Abbreviations

HEP: home exercise program
mHealth: mobile health
PICO: Population, Intervention, Comparison, Outcome
PRISMA-ScR: Preferred Reporting Items for Systematic Reviews and Meta-Analyses Extension for Scoping Reviews

## Data Availability

Datasets will be presented in the scoping review and made available from the corresponding author upon reasonable request.
