# Supplementary material for: Gamification in mHealth Apps for Rehabilitation: Protocol for a Scoping Review
Source: JMIR Res Protoc. 2025 Apr 28;14:e63600. doi: 10.2196/63600 (PMC12070003; doi:10.2196/63600)
Supplement: Multimedia Appendix 1 [file resprot_v14i1e63600_app1.docx]

Multimedia Appendix 1: Search Strategy

MEDLINE (Ovid)

| Number | Search items | Records retrieved |
| --- | --- | --- |
| 1 | exp rehabilitation/ or exp telerehabilitation/ [MeSH Terms] | 353,713 |
| 2 | (rehabilitation or telehealth or physical therapy or physiotherapy or pain management or preoperative care or postoperative care or disease management or patient education).mp. [mp= title, abstract, original title, name of substance word, subject heading word, floating sub-heading word, keyword heading word, organism supplementary concept word, protocol supplementary concept word, unique identifier, synonyms] | 700,240 |
| 3 | 1 or 2 | 895,112 |
| 4 | exp video games/ [MeSH Terms] | 7,341 |
| 5 | (game* or gami* or exergame* or serious game or virtual or augmented reality or mHealth or eHealth or remote or mobile or wearable or web* or video* or digital*).mp. [mp= title, abstract, original title, name of substance word, subject heading word, floating sub-heading word, keyword heading word, organism supplementary concept word, protocol supplementary concept word, unique identifier, synonyms] | 836,499 |
| 6 | 4 or 5 | 836,506 |
| 7 | exp Exercise therapy/ or exp Physical therapy modalities/ [MeSH Terms] | 178,953 |
| 8 | (therapeutic exercise or exercise* or physical training or physical exercise or resistance training or exercise therapy or kinesiology or manual therapy or kinesiotherapy or aerobic exercise).mp. [mp= title, abstract, original title, name of substance word, subject heading word, floating sub-heading word, keyword heading word, organism supplementary concept word, protocol supplementary concept word, unique identifier, synonyms] | 421,440 |
| 9 | 7 or 8 | 524,466 |
| 10 | 3 and 6 and 9 | 11,842 |
| 11 | Limit to human studies and full-text | 2,148 |
